# The INCH-trial: a multicenter randomized controlled trial comparing short- and long-term outcomes of open and laparoscopic surgery for incisional hernia repair

**DOI:** 10.1007/s00464-023-10446-7

**Published:** 2023-10-09

**Authors:** Nadine van Veenendaal, Marijn Poelman, Jan Apers, Huib Cense, Hermien Schreurs, Eric Sonneveld, Susanne van der Velde, Jaap Bonjer

**Affiliations:** 1 Department of Surgery, Amsterdam University Medical Center, Boelelaan 1117, 1081 HV Amsterdam, The Netherlands; 2 Department of Anesthesiology, University Medical Center, Groningen, The Netherlands; 3 Department of Surgery, Franciscus Gasthuis & Vlietland, Rotterdam, The Netherlands; 4 Department of Surgery, Red Cross Hospital, Beverwijk, The Netherlands; 5 Department of Surgery, Northwest Clinics, Alkmaar, The Netherlands; 6 Department of Surgery, Dijklander Hospital, Hoorn, The Netherlands

**Keywords:** Incisional hernia, Laparoscopic repair, Open repair, Quality of life, Abdominal wall, RCT

## Abstract

**Background:**

Laparoscopic incisional hernia repair is increasingly performed worldwide and expected to be superior to conventional open repair regarding hospital stay and quality of life (QoL). The INCisional Hernia-Trial was designed to test this hypothesis.

**Methods:**

A multicenter parallel randomized controlled open-label trial with a superiority design was conducted in six hospitals in the Netherlands. Patients with primary or recurrent incisional hernias were randomized by computer-guided block-randomization to undergo either conventional open or laparoscopic repair. Primary endpoint was postoperative length of hospital stay in days. Secondary endpoints included QoL, complications, and recurrences. Patients were followed up for at least 5 years.

**Results:**

Hundred-and-two patients were recruited and randomized. In total, 88 patients underwent surgery and were included in the intention-to-treat analysis (44 in the open group, 44 in the laparoscopic group). Mean age was 59.5 years, gender division was equal, and BMI was 28.8 kg/m. The trial was concluded early for futility after an unplanned interim analysis, which showed that the hypothesis needed to be rejected. There was no difference in primary outcome: length of hospital stay was 3 (range 1–36) days in the open group and 3 (range 1–12) days in the laparoscopic group (*p* = 0.481). There were no significant between-group differences in QoL questionnaires on the short and long term. Satisfaction was impaired in the open group. Overall recurrence rate was 19%, of which 16% in the open and 23% in the laparoscopic group (*p* = 0.25) at a mean follow-up of 6.6 years.

**Conclusions:**

In a randomized controlled trial, short- and long-term outcomes after laparoscopic incisional hernia repair were not superior to open surgery. The persisting high recurrence rates, reduced QoL, and suboptimal satisfaction warrant the need for patient’s expectation management in the preoperative process and individualized surgical management.

**Trial registration:**

Netherlands Trial Register NTR2808.

**Supplementary Information:**

The online version contains supplementary material available at 10.1007/s00464-023-10446-7.

Minimally invasive techniques have revolutionized the field of surgery with benefits that include reduced postoperative pain, earlier return to normal activities, and fewer postoperative complications compared to open techniques [1]. Despite the increased application of minimally invasive surgery, incisional hernias remain a common complication in surgery with incidences of 6–12% [2, 3]. Pain, discomfort, and reduced quality of life (QoL) are the main symptoms in 78–85% of patients undergoing incisional hernia repairs [4–6].

Over the last three decades, laparoscopic repair has gained popularity in the management of incisional hernias. Laparoscopic incisional hernia repair is believed to be superior in terms of postoperative recovery time, wound infections, and recurrence rates [7]. Although proven to be safe [8], to date there is inconclusive evidence that laparoscopic incisional hernia repair is superior to conventional open repair [9] in terms of operative time, length of hospital stay, complications, pain, or QoL [10, 11].

In light of the ongoing debate about the expected merits of laparoscopic versus open incisional hernia repair, several randomized controlled trials (RCTs) have been conducted in the last two decades [8, 12–16]. However, the majority of these RCTs were conducted in the first two decades after the introduction of laparoscopic incisional hernia repair [17], and length of follow-up did not extend more than 35 months [15]. Primary endpoints have often been surgery-specific outcomes, whereas more recently the focus has shifted toward patient-centered outcomes. Since the goal of surgical incisional hernia repair is to improve QoL on the long term, this prompted the need for a randomized controlled trial.

In view of increasing value of patient-centered outcomes and the scarcity of long-term outcomes after incisional hernia repair, we aimed to conduct a trial with focus on long-term follow-up. The INCisional Hernia-Trial (INCH-Trial) was designed to compare the short- and long-term outcomes of laparoscopic and open incisional hernia repair.

## Materials and methods

### Study design

We conducted a multicenter parallel randomized controlled open-label trial comparing the short- and long-term outcomes of conventional open and laparoscopic surgery for incisional hernia repair. A complete overview of the study protocol has been published previously [18]. In summary, adult patients who were referred to the surgical clinic for assessment of a primary or recurrent incisional hernia were eligible for participation. Exclusion criteria were pregnancy, abdominal ostomy, history of open abdomen treatment, unable to give informed consent, a life expectancy of less than 1 year, an immune-compromised status, and ASA > 3. The trial was registered with the Netherlands Trial register (NTR2808). The CONSORT 2010 guidelines were used to report the outcomes of this study [19].

Patients were enrolled at the outpatient clinic of six hospitals in The Netherlands (Amsterdam University Medical Center, Medical Center Leeuwarden, Northwest Clinics, Red Cross Hospital, Slotervaart Medical Center, Dijklander Hospital). The participating centers comprised two surgeons per center being hernia experts responsible for enrolling, randomizing, and operating patients. Approval was obtained of the Medical Ethical Committee in all participating hospitals. Informed written consent was obtained from all the patients before inclusion in the trial. An independent data and safety monitoring board was not installed, since both surgical repair methods had proven to be safe.

Randomization was performed using a computer-generated program. Eligible patients were randomly assigned in a 1:1 ratio to undergo open or laparoscopic repair. An internet application allowed central randomization.

The employed open repair method was at the discretion of the participating surgeon. Onlay, sublay as well as component separation technique were allowed as long as a mesh was used with an overlap of at least 5 cm. Laparoscopic repair of incisional hernias entailed employment of a mesh intraperitoneal with an overlap of the defect of 3 to 5 cm. The choice for sutures or tackers, the exact fixation repair method, and closure of the fascia defect were at the discretion of the surgeon. Postoperatively, details of the surgical repair method were entered in an online database.

### Outcomes

Primary outcome was length of hospital stay, which was defined as the time until discharge home. Criteria for discharge were that patients had to move normally, tolerate a normal diet, and have a NRS < 5 without the use of opiates. It was upon the discretion of the surgeon whether these criteria were met.

Secondary outcomes were QoL, re-operation rates, recurrence rates, and 28 days post-surgery mortality. The SF-36 and Carolina Comfort Scale (CCS) were used as QoL measures. The SF-36 is a validated, wide-spread, generic, frequently used QoL questionnaire covering eight health domains and is available in Dutch [20, 21]. The CCS is a validated, disease-specific questionnaire for patients after incisional hernia repair containing 23 items [22, 23]. The CCS has been proven to be valuable in hernia research and has been validated for the Dutch language [24].

Baseline characteristics were registered at the outpatient clinic. Patient-related factors were age, sex, BMI, ASA-classification, previous abdominal surgeries, co-morbidity, smoking, and family history for abdominal wall hernias. Hernia-related factors were primary or recurrent incisional hernia, meshes in situ, previous radio diagnostics, and the EHS-classification [25]. Postoperative complications were categorized according to the Clavien-Dindo classification [26]. Two weeks post-surgery daily NRS scores, use of analgesic, morbidity, and mortality were recorded. At 3 months, 3 years, and 5 years follow-up, QoL, recurrence rates and morbidity were assessed.

Due to interest in both short- and long-term outcomes, patients were followed up for 5 years. Due to the COVID-19 pandemic from March 2020 on and hospital visiting restrictions for research purposes, patients could not be invited for physical examination at the outpatient clinic. Instead, patients were contacted by telephone and video calling.

### Statistical analysis

The INCH-trial had a superiority design, hypothesizing that length of hospital stay would be shorter after laparoscopic repair. The required sample size was calculated based on an average hospital stay of 2 days (SD 5) after laparoscopic repair [11]. A difference of two or more days in favor of the laparoscopic group was considered a significant difference. The sample size accordingly required 135 patients in each treatment arm (alpha 0.05/power 0.9). Compensating for loss to follow-up, we planned to enroll 300 participants in total.

Statistical analyses were carried out using IBM SPSS Statistics v23. Continuous variables were tested with Student *t* test or Mann–Whitney test in case of, respectively, normal and nonnormal distribution. Categorical variables were tested using Chi-square statistics in case of normal distribution and expected values above 5. Fisher’s exact tests were used for categorical variables when expected values in any of the cells of the contingency table were below 5. Differences in primary and secondary outcomes of both intervention groups were calculated, as well as their 95% confidence intervals. Statistical tests with *p* = 0.05 were considered significant. Analyses were carried out according to the intention-to-treat principle. Patients without an intervention were excluded to prevent type II error [27].

### Interim-analysis

In March 2017, an unplanned interim analysis was conducted on the basis of data from all randomly assigned patients of whom the primary outcome was known. The study protocol had been written and approved in 2012, and since the beginning of the study, developments in incisional hernia repair methods had changed daily practice. The laparoscopic technique used in the INCH-trial without closure of the defect was getting less popular, and there was a shift toward the use of the robot, reconstruction of abdominal wall layers, and a retromuscular mesh position.

Due to the variety in techniques and inconclusive evidence which repair method is superior, the research group thought an interim analysis was needed. Results indicated a probability of more than 95% that we would find no significant difference in hospital stay if we would include more patients in the trial. After considering all the evidence, the research group decided early closure of the trial for futility. Owing to the early stopping of the trial, the primary efficacy analyses and secondary efficacy analyses included all patients who had underwent incisional hernia repair. End date of the trail was April 1st 2017.

## Results

### Patients

**Fig. 1:**
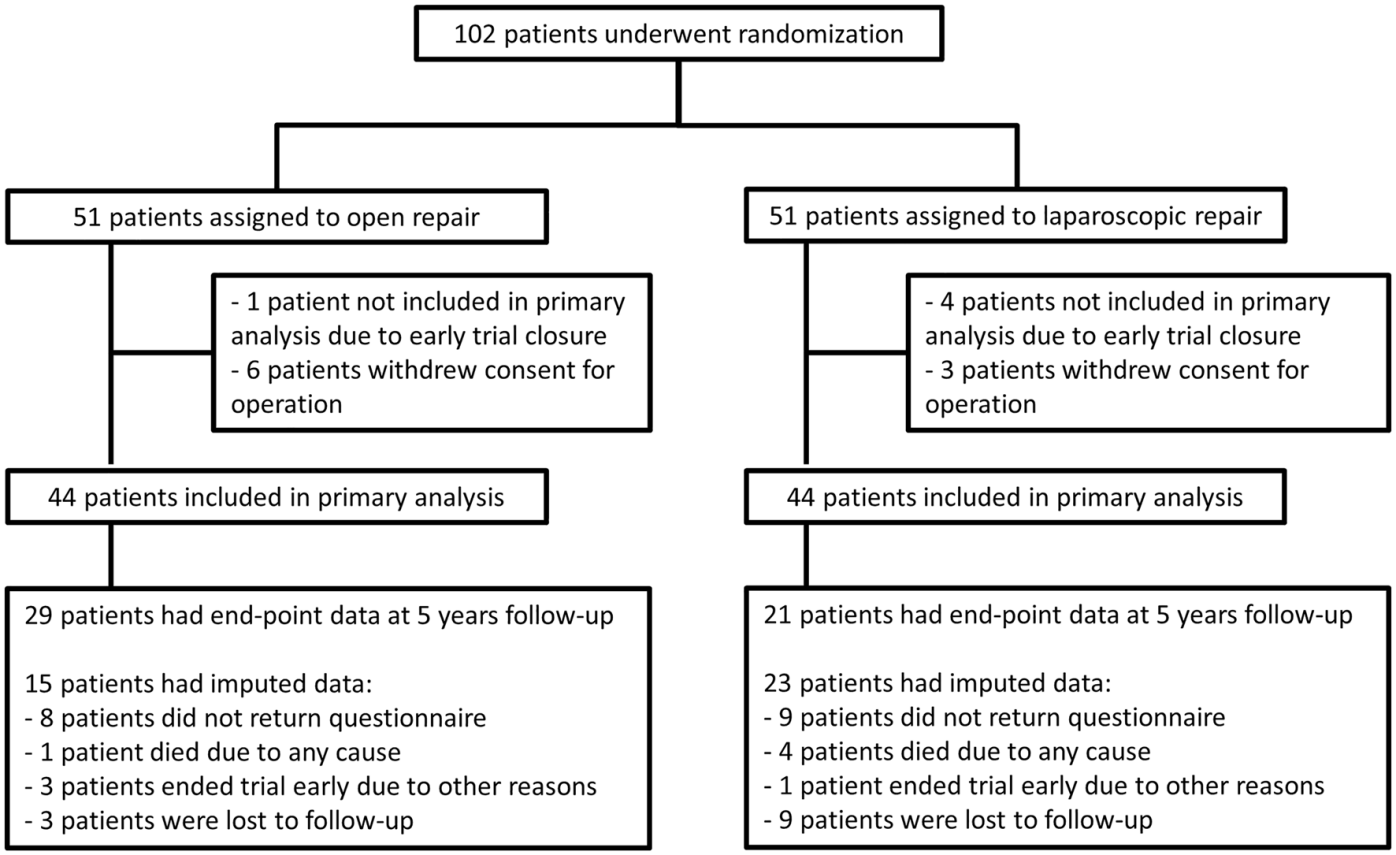
CONSORT flow chart of patients

From October 2012 until April 2017, a total of 102 patients with incisional hernias underwent randomization. Of these patients, 51 were assigned to undergo conventional open surgery and 51 to undergo laparoscopic repair. After drop out of 14 patients following randomization, 88 patients remained (Fig. 1). Eighty-eight patients were included for analysis (44 in the open group, 44 in the laparoscopic group), with a mean age of 59.5 years, an equal gender division, and BMI of 28.8 kg/m. The baseline characteristics of the patients were similar in both groups (Table 1). The majority of the patients had a primary hernia (95%) and one abdominal operation in the medical history (52%). Most hernias were smaller than 10 cm (82%) and located either in the epigastric (41%) or umbilical (38%) region. Perioperative details are summarized in Table 2.

**Table 1:** Baseline characteristics of the patients

	Total (*n* = 88)	Open repair (*n* = 44)	Laparoscopic repair (*n* = 44)	*p* value
Gender (Female)	44 (50%)	18 (41%)	26 (59%)	0.090
Age at operation, mean (range)	59.5 (12.1)	58.03 (29.89–80.81)	60.90 (35.54–81.05)	0.268
BMI in kg/m^2^, mean (SD)	28.83 (4.49)	28.83 (4.33)	28.84 (4.39)	0.991
ASA class (*n* (%))				
1	16 (18%)	10 (23%)	6 (14%)	0.928
2	51 (58%)	25 (57%)	26 (59%)	0.422
3	21 (24%)	9 (20%)	12 (27%)	0.175
Smoking status (*n* (%))				
Yes	20 (23%)	9 (20%)	11 (25%)	0.884
No	49 (56%)	23 (53%)	26 (59%)	
Missing	19 (20%)	12 (27%)	7 (16%)	
Hernia frequency				
Primary incisional hernia repair	84 (95%)	40 (91%)	44 (100%)	0.041
Recurrent incisional hernia repair	4 (5%)	4 (9%)	0	
Previous abdominal operations				
1	46 (52%)	20 (45%)	26 (59%)	0.112
2	14 (16%)	4 (9%)	10 (23%)	
3	12 (14%)	9 (20%)	3 (7%)	
> 4	4 (5%)	3 (7%)	1 (2%)	
Missing	12 (14%)	8 (18%)	4 (9%)	
Hernia classification				
W1 (< 4 cm)	37 (42%)	19 (43%)	18 (41%)	0.946
W2 (4–10 cm)	35 (40%)	17 (39%)	18 (41%)	
W3 (> 10 cm)	15 (17%)	8 (18%)	7 (16%)	
Missing	1 (1%)	0	1 (2%)	
Hernia location				
M1 subxiphoidal	1 (1%)	1 (2%)	0	0.936
M2 epigastric	36 (41%)	16 (36%)	20 (45%)	
M3 umbilical	33 (38%)	18 (41%)	15 (34%)	
M4 infraumbilical	4 (5%)	2 (5%)	2 (5%)	
L1 subcostal	2 (2%)	1 (2%)	1 (2%)	
L2 flank	3 (3%)	1 (2%)	2 (5%)	
L3 iliac	6 (7%)	3 (7%)	3 (7%)	
Modality of hernia detection				
Clinical	36 (41%)	15 (34%)	21 (48%)	0.231
Ultrasound	19 (20%)	13 (30%)	6 (14%)	
CT	30 (68%)	15 (34%)	15 (34%)	
MRI	1 (1%)	1 (2%)	0	
US + CT	2 (2%)	0	2 (5%)

**Table 2:** Perioperative outcomes

	Total (*n* = 88)	Open repair^a^ (*n* = 44)	Laparoscopic repair^b^ (*n* = 44)	*p* value
Duration of operation, min (mean, SD)	76.75 (38.99)	79.97 (46.13)	73.20 (29.63)	0.496
Change of surgical technique^c^, *n* (%)	7 (8%)	3 (6.8%)	4 (9.1%)	0.347
Mesh use, *n* (%)				
No mesh	5 (6%)	4 (9%)	1 (2%)	n.a
Parietex mesh (medtronic)	34 (39%)	11 (25%)	23 (53%)	
Symbotex (medtronic)	1 (1%)	0	1 (2%)	
Marlex	3 (3%)	3 (7%)	0	
Ultrapro (ethicon)	6 (7%)	5 (11%)	1 (2%)	
Physiomesh (ethicon)	8 (9%)	3 (7%)	5 (11%)	
Prolene (ethicon)	9 (10%)	8 (18%)	1 (2%)	
Ventralex (BD)	3 (3%)	2 (5%)	1 (2%)	
Other mesh	5 (6%)	2 (5%)	3 (7%)	
Unknown	14 (%)	6 (14%)	8 (18%)	
Mesh location, *n* (%)				
Onlay		4 (9%)		n.a
Sublay		33 (75%)		
Nonspecified		3 (7%)		
Intraperitoneally			43 (98%)	
No mesh		4 (9%)	1 (2%)	
Fixation method, *n* (%)				
None	1 (1%)	1 (2%)	0	n.a
Sutures	21 (24%)	18 (41%)	3 (7%)	
Tackers	32 (36%)	4 (9%)	28 (64%)	
Glue	1 (1%)	1 (2%)	0	
Missing/unknown	33 (38%)	20 (45%)	13 (30%)	
Antibiotics, *n* (%)				
None	20 (23%)	10 (23%)	10 (23%)	0.600
Antibiotics	68 (77%)	34 (77%)	34 (77%)	
Wound drain, *n* (%)				
Yes	10 (11%)	9 (20%)	1 (2%)	n.a
No	35 (40%)	8 (18%)	27 (61%)	
Missing/unknown	45 (51%)	27 (61%)	16 (36%)	

^a^Concomitant procedures during open repair: in 1 case, an umbilical hernia repair was performed

^b^Concomitant procedures during laparoscopic repair: in 1 case an umbilical hernia repair, in 1 case a cholecystectomy, and in 1 case a cholecystectomy and umbilical hernia repair

^c^The number of cases in which was decided preoperatively to switch from open to laparoscopic repair, or intra-operatively conversion from laparoscopic to open repair

Complication-free recovery occurred in 22 of 44 (50%) patients in the open group, and in 21 of 44 (48%) patients in the laparoscopic group. Seromas, wound infections, and wound dehiscence were the most frequent complications (Table 3).

**Table 3:** Short-term postoperative outcomes

	Open repair (*n* = 44)	Laparoscopic repair (*n* = 44)	*p* value
Length of hospital stay, days (median, range)	3 (range 1–36)	3 (range 1–12)	0.481
Clavien–Dindo grade and detailed complications, number (%)			
No complication	22 (50%)	21 (48%)	n.s
Grade I	6 (14%)	10 (23%)	n.s
Superficial surgical site infection	3	2	
Seroma	3	6	
Hematoma	0	2	
Grade II	4 (9%)	4 (9%)	n.s
Pneumonia	1	3	
Ileus	2	1	
Ischemic heart disease	1	0	
Deep surgical site infection	0	0	
Grade III			
Wound dehiscence	2 (5%)	3 (7%)	n.s
Grade IV			
Sepsis, re-operation and ICU-admission	1 (2%)	1 (2%)	n.s
Grade V			
Death	0	0	n.s
Missing/unknown	13 (30%)	10 (23%)	
Mortality at 28 days follow-up, number (%)	0	0	n.a

Mean follow-up times for the QoL questionnaires were 97 (range 36–147) days at 3 months follow-up and 6.6 years for long-term follow-up (range 4.3–8.7 years). Response rate for the long-term follow-up was 76%, accounting for deceased patients. At long-term follow-up, 50 of 88 (57%) patients completed the questionnaires. Twenty-one of 88 (24%) patients were lost-to-follow-up during varying reasons (Fig. 1).

### Primary outcomes

Length of hospital stay was 3 (range 1–36) days in the open group and 3 (range 1–12) days in the laparoscopic group (*p* = 0.481) (Table 3).

**Fig. 2:**
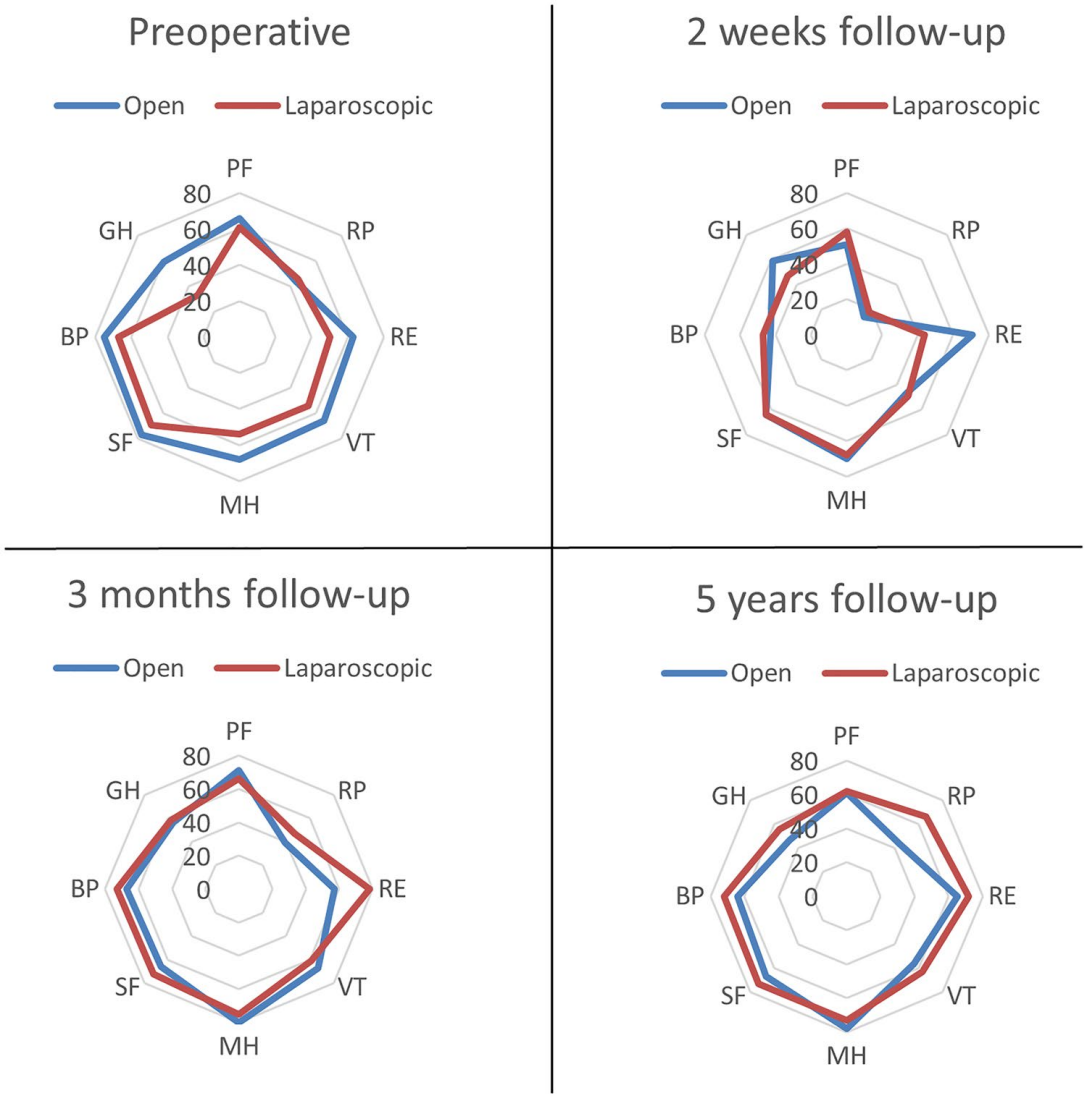
SF-36 at different follow-up timepoints for the open and laparoscopic group. *PF* physical functioning, *RP* role physical, *RE* role emotional, *VT* vitality, *MH* mental health, *SF* social functioning, *BP *bodily pain

The SF-36 scores are summarized per group over time (Table 4 and Fig. 2). No significant differences in SF-36 subscales were detected between the two intervention groups at any point of follow-up. Condensed data from the SF-36 showed a tendency toward general improvement at 3 months follow-up for the laparoscopic group. A tendency toward general improvement is seen as reflected by the subscale health change of the SF-36 for both groups at 3 months follow-up.

**Table 4:** Pre- and postoperative SF-36 scores (mean, SD)

SF-36 item	Preoperatively (*n* = 88)	3 months follow-up (*n* = 88)	5 years follow-up (*n* = 50)
Physical functioning (PF)			
Open	65.9 (23.6)	70.7 (22.4)	61.1 (27.7)
Laparoscopic	60.8 (21.5)	66.1 (22.6)	62.3 (29.5)
* p* value	0.567	0.691	0.892
Role limitations due to physical health (RP)			
Open	43.8 (37.1)	39.3 (45.3)	44.2 (45.4)
Laparoscopic	45.8 (36.7)	47.2 (45.8)	65.8 (44.3)
* p* value	0.884	0.735	0.119
Role limitations due to emotional problems (RE)			
Open	62.5 (43.7)	57.1 (46)	65.4 (43.8)
Laparoscopic	50 (46.1)	77.8 (33.3)	71.9 (42.0)
* p* value	0.471	0.315	0.617
Energy/fatigue/vitality (VT)			
Open	65.6 (14.1)	67.1 (16.0)	55.5 (25.8)
Laparoscopic	53.8 (20.6)	60.6 (13.1)	63.1 (20.0)
* p* value	0.077	0.380	0.289
Emotional well-being/mental health (MH)			
Open	68.2 (19.4)	80 (15.7)	77.5 (17.8)
Laparoscopic	53.5 (22.8)	75.1 (16.0)	73.1 (18.0)
* p* value	0.073	0.550	0.403
Social functioning (SF)			
Open	76.5 (18.7)	66.1 (33.6)	67.0 (35.6)
Laparoscopic	69.2 (23.1)	72.2 (29.2)	73.0 (22.5)
* p* value	0.356	0.701	0.514
Bodily pain (BP)			
Open	75.3 (22.9)	67.1 (27.6)	64.4 (31.3)
Laparoscopic	66.7 (30.8)	72.8 (21.5)	71.7 (26.4)
* p* value	0.395	0.652	0.406
General health (GH)			
Open	58.8 (29.2)	55.9 (22.4)	47.2 (26.4)
Laparoscopic	33.3 (26.9)	58.3 (11.2)	55.6 (25.1)
* p* value	0.202	0.784	0.281
Health change (HC)			
Open		67.9 (34.5)	38.4 (17.3)
Laparoscopic		66.7 (30.6)	46.1 (20.9)
* p* value		0.943	0.178

The open group has lower scores on all domains except mental health at 5 years follow-up. However, the differences between the two groups were not significant. Although not significantly, general health is scored lower by the open group at 5 years follow-up.

**Fig. 3:**
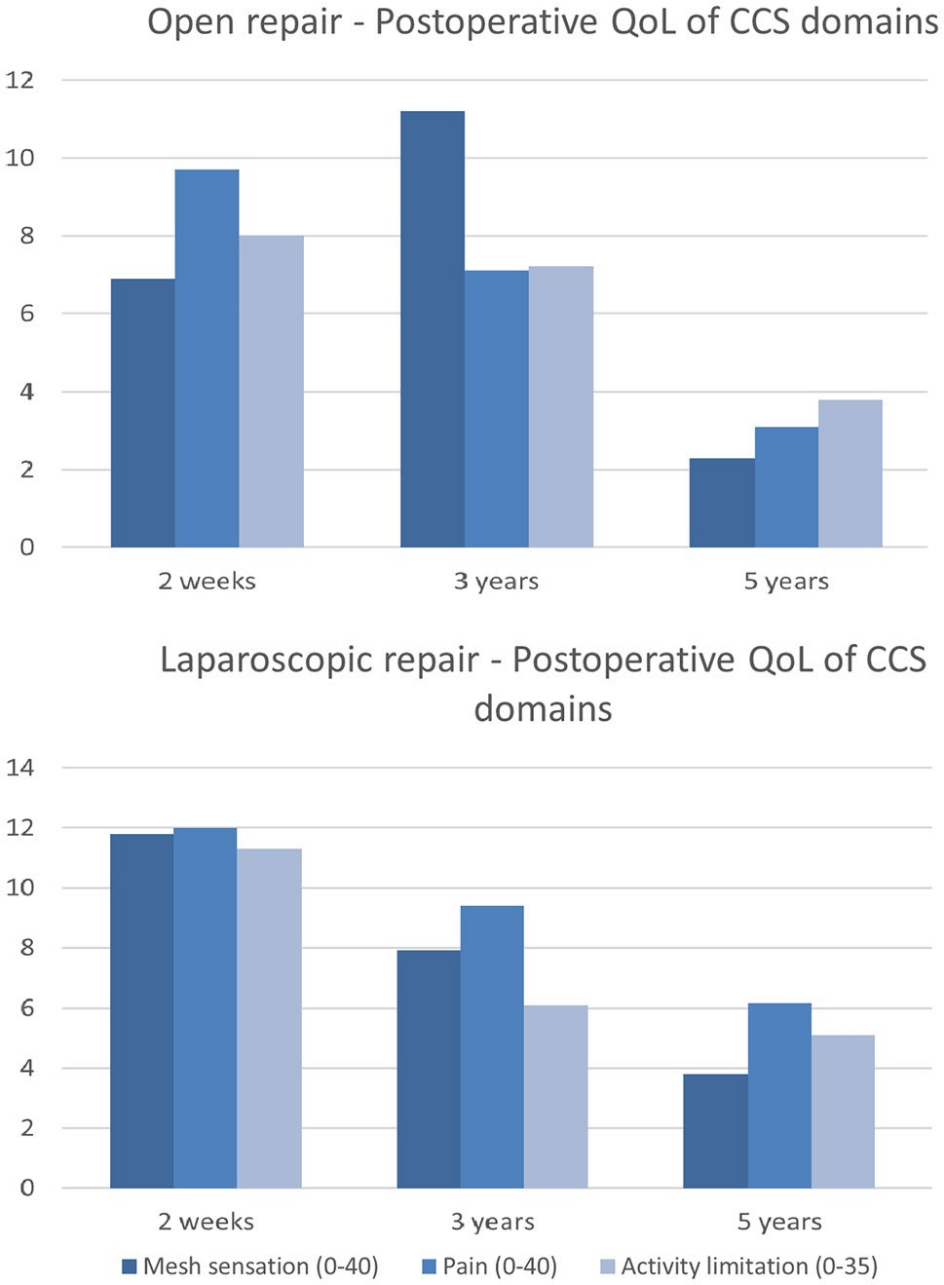
Postoperative Quality of Life (QoL) scores of different Carolina Comfort Scale (CCS) domains for open and laparoscopic incisional hernia repair at different times in follow-up

No significant differences in CCS were found at any time of follow-up between the two groups (Fig. 3, Online Supplements 1–3). All scores in the three CCS domains were higher in the open group at 2 weeks and 5 years follow-up; however, compared with the laparoscopic group, these differences were not significant. Patients were considered symptomatic if the total score of a CCS domain exceeded 1 [22]. The total number of symptomatic patients according to the CCS is remarkably high, with 89%, 83%, and 35% at, respectively, 2 weeks, 3 months, and long-term follow-up. However, significant differences in symptomatic patients were not observed between the two groups.

### Secondary outcomes

Perioperative details and postoperative complications are summarized in Table 2. At 28 days post-surgery, all patients were still alive. At 1-year follow-up, one patient had died, and at 3 years follow-up, two patients had died. At 5-year follow-up, one patient in the open group and four patients in the laparoscopic group had died. Cause of death in patients who died during the follow-up period was not associated with the incisional hernia repair.

**Fig. 4:**
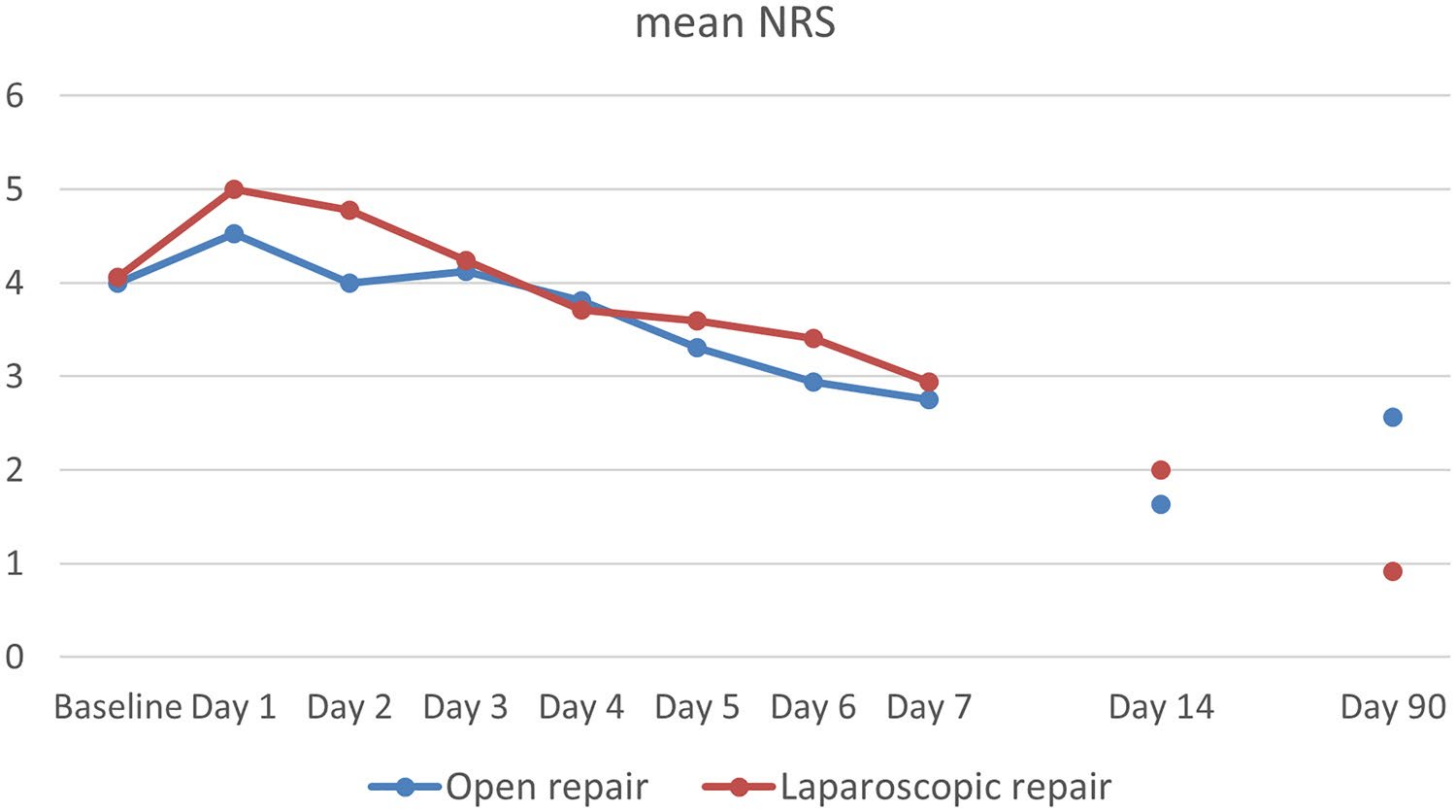
Mean daily NRS scores on a 0–10 scale preoperatively (baseline) and in the postoperative period

Mean preoperative NRS was 4.0 (SD 2.97) in the open and 4.1 (SD 2.25) in the laparoscopic group (*p* = 0.95). The mean NRS was 4.5 (SD 2.55) and 5.0 (SD 2.81) on the first day postoperatively, and decreased to a mean of 2.75 (SD 1.95) and 2.94 (SD 2.36) in the open and laparoscopic group, respectively (*p* = 0.80) at 7 days follow-up. NRS scores further decreased at 14 days follow-up (Fig. 4). Although not significantly, higher NRS scores were observed in the open group at 3 months follow-up. At 5 years follow-up, NRS scores were 1.86 (SD 2.90) and 1.68 (SD 2.77) in the open and laparoscopic group (*p* = 0.84).

In total, a recurrence occurred in thirteen out of 68 (19%) patients at a mean follow-up of 6.6 years (range 4.3–8.7 years). Six of 37 (16%) patients in the open group, and seven of 31 (23%) patients in the laparoscopic group had a recurrence (*p* = 0.25) (Online Supplement 4). Due to COVID-19 visiting restrictions in all participating hospitals, all patients underwent follow-up by telephone or video calling.

At long-term, follow-up patients were interviewed about the level of satisfaction after incisional hernia repair. In total, thirteen of fifty (26%) patients were not satisfied with the result of their incisional hernia repair. Twenty-three of fifty (46%) patients thought the overall status of their abdominal wall was either similar or worse as compared with the situation before surgical repair. Seven of fifty (14%) patients would not undergo the same operation again, and eleven of fifty (22%) patients would not recommend the operation. The majority of patients who were not satisfied at 5 years follow-up had undergone an open incisional hernia repair in the past (Table 5).

**Table 5:** Satisfaction after incisional hernia surgery

Question	Total (*n* = 50)	Open repair (*n* = 29)	Laparoscopic repair (*n* = 21)	*p* value
Are you satisfied with the result?				
Yes, *n* (%)	37 (74%)	19 (66%)	18 (86%)	0.108
No, *n* (%)	13 (26%)	10 (34%)	3 (14%)	
Overall status of the abdominal wall compared with the situation before the repair? *n* (%)				
Better, *n* (%)	27 (54%)	15 (52%)	12 (57%)	0.069
Similar, *n* (%)	11 (22%)	4 (14%)	7 (33%)	
Worse, *n* (%)	12 (24%)	10 (34%)	2 (10%)	
Would you undergo incisional hernia repair again? *n* (%)				
Yes, *n* (%)	43 (86%)	23 (79%)	20 (95%)	0.115
No, *n* (%)	7 (14%)	6 (21%)	1 (5%)	
Would you recommend this operation to others? *n* (%)				
Yes, *n* (%)	39 (78%)	19 (66%)	20 (95%)	0.012
No, *n* (%)	11 (22%)	10 (34%)	1 (5%)	

## Discussion

This randomized controlled trial showed that laparoscopic incisional hernia repair was not superior to conventional open repair for length of hospital stay. Median length of hospital stay was similar in both groups, with 3 days in both the open and laparoscopic group. There were no significant between-group differences in QoL at short- and long-term follow-up. A remarkably high number of patients were symptomatic according to the CCS having ‘mild but bothersome’ to ‘disabling’ pain, with 89%, 83%, and 35% at, respectively, 2 weeks, 3 months, and long-term follow-up. A third of the patients in the open group was not satisfied at long-term follow-up compared to fifteen percent in the laparoscopic group, and would not go for this operation again.

The results of this study do not change the ongoing debate of conventional open or laparoscopic surgical repair. Although laparoscopic techniques have become the standard of care for many operations, convincing evidence to conclude that laparoscopic incisional hernia repair is superior to conventional open repair is lacking. Laparoscopic incisional hernia repair is found to lead to significantly fewer wound infections as compared with open approach, but superiority for other outcomes is still not proven [7, 9].

However, the laparoscopic technique used in the INCH-trial is getting less popular. In the last years, there is a shift toward the use of the robot in abdominal wall surgery [28]. With the use of the robot the abdominal wall layers can be reconstructed and the midline is repaired. Furthermore, the mesh is placed in the retromuscular position, avoiding contact between viscera and the mesh, that might cause less adhesions.

Length of hospital stay is assumed to be shorter in laparoscopic incisional hernia repair [7, 29]. However, our results showed no significant differences in hospitalization. A meta-analysis of 11 RCTs found a significantly shorter hospital stay in patients undergoing laparoscopic hernia repair [29]. Both ventral and incisional hernias were included, potentially challenging the external validity to incisional hernias alone. Two RCTs on incisional hernia repair showed no difference in length of hospital stay [15, 16], which is in line with the results of this study. Hospital stay in the INCH-trial was 1 day longer than reported in another RCT [15]. The inclusion of almost 60% larger hernias (W2 and W3) can be a possible explanation.

QoL was comparable for both groups at short- and long-term follow-up. Only a trend of better QoL outcomes in the laparoscopic group was observed, but scores differed not significantly. Improvement of QoL was mostly reflected in the physical subscales of the SF-36 instrument, which is in line with previous studies [30]. Better short-term QoL after laparoscopic repair has been described previously [16]. However, the same study showed no differences in QoL between laparoscopic and open repair on the long term. Although guidelines of the International Endohernia Society recommend laparoscopic repair above open repair when considering QoL [7], the results of the INCH-trial cannot confirm this recommendation. Comparable QoL between open and laparoscopic incisional hernia repair was also found in other studies, with follow-ups of 1 and 4 years [31, 32]. The longest follow-up until now also showed comparable QoL between open and laparoscopic repair at almost 13 years follow-up [33].

In this study, two QoL questionnaires were used. The SF-36 is a generic instrument and the CCS is a widely accepted, disease-specific QoL instrument validated in all hernia types with mesh repair. Although QoL improved according to the SF-36, the CCS showed that a significant group of patients experienced ‘mild to bothersome pain’ at different timepoints of follow-up. Besides, 26% of the patients were not satisfied and 46% thought the abdominal wall was similar or worse compared to before the operation at long-term follow-up. Therefore, the question whether or not a patient would undergo incisional hernia repair again knowing the results of the operation is characteristic for the level of satisfaction.

QoL questionnaires seem to vary in their outcomes making interpretation of the results rather difficult. Given the huge variety in different assessment measures used in hernia research [34], standardization will benefit future research. Additionally, we need to involve patients in the decision-making process. Patient’s expectations need to be part of preoperative counseling and evaluations of PROs after incisional hernia repair are relevant for measuring the rate of success.

Overall recurrence rate at long-term follow-up was 19%, of which 16% in the open and 23% in the laparoscopic group (*p* = 0.25). Other studies have reported similar recurrence rates of 14% in the open group and 18% in the laparoscopic group [15]. A recurrence rate of 21% at 10 years follow-up was reported in another study, suggesting that our recurrence rate is in line with the known incidence. The number of patients was too small to identify associations with hernia size of location.

The high incidence and high recurrence rate make incisional hernias the most common complication after abdominal wall surgery. The majority of patients with incisional hernias develop symptoms such as pain, discomfort or cosmetic complaints. Incisional hernias can be invalidating for patients, and the QoL in these patients as well as their chances for employment are reduced. Approximately, eighty percent of the patients with an incisional hernia undergo surgical repair [5]. Therefore, research into incisional hernias remains of utmost importance.

This study should be seen in light of several limitations. First of all, the design of this trial can be considered a limitation. A randomized controlled trial with a superiority design is the most optimal study design to determine if one surgical intervention is superior to the prevailing one. However, the likelihood to find a minimal clinically important difference is challenging [35]. Therefore, noninferiority RCT designs have gained popularity in several fields [36, 37]. Hence, evaluation of trial design in surgery is warranted [38].

Another limitation was the evolution of new surgical techniques during the trial, with promising results [27]. Although laparoscopic repair of incisional hernias was new and had been proven safe, focus shifted to reconstruction of the midline and to mesh placement outside the abdominal cavity with or without the use of the robot. Parallel to the design and the conduction of the study, these minimally invasive repair methods with the use of a robot were developed, rendering the laparoscopic technique used in the INCH-trial less popular.

The high level of heterogeneity in surgical procedures in the INCH-trial can be considered another limitation. The variety in surgical techniques makes it difficult to compare all the cases. However, with the design of this study, we aimed for an adequate reflection of daily practice.

The potential bias and loss of statistical power due to the loss of follow-up can be considered another limitation of this study. Response rates at 5 years follow-up were 78% for recurrence rate evaluation and 57% for QoL questionnaires. Although a cut-off of 80% follow-up is ideal in Evidence-Based Medicine (EBM) [39], rates of 50–80% follow-up have been suggested as acceptable [40]. Response rates are influenced by many amendable factors. Loss to follow-up in this study was inevitable for the long-term outcomes, but the current response rates seem to be reasonable.

Lastly, the hospital visiting restrictions due to the COVID-19 pandemic were a limitation for the follow-up of this study. Unfortunately, physical examination could not be conducted, potentially resulting in missing recurrences. Awareness about this limitation elicited more extensive interviews to complete thorough follow-up. However, the high number of reported symptoms at long-term follow-up is suggestive for an underestimation of the actual recurrence rates.

## Conclusion

In this randomized controlled trial, short- and long-term outcomes after laparoscopic incisional hernia repair were not superior to open surgery. Reconstruction of the midline and retromuscular mesh placement (minimally invasive or open) is currently considered best practice, and therefore, the laparoscopic intraperitoneal mesh placement is only used in selected cases (i.e., morbid obese patients). The persisting high recurrence rates, reduced quality of life, and suboptimal satisfaction warrant the need for patient’s expectation management in the preoperative process and individualized surgical management.

### Supplementary Information

Below is the link to the electronic supplementary material.

Supplementary file1 (DOCX 23 KB)

Supplementary file2 (DOCX 22 KB)

Supplementary file3 (DOCX 22 KB)

Supplementary file4 (DOCX 21 KB)
