# Supplementary material for: The INCH-trial: a multicenter randomized controlled trial comparing short- and long-term outcomes of open and laparoscopic surgery for incisional hernia repair
Source: Surg Endosc. 2023 Oct 9;37(12):9147–58. doi: 10.1007/s00464-023-10446-7 (PMC10709221; doi:10.1007/s00464-023-10446-7)
Supplement: Supplementary file 1 — Supplementary file1 (DOCX 23 KB) [file 464_2023_10446_MOESM1_ESM.docx]

**SUPPLEMENT**

| **Supplement 1. Postoperative quality of life score by CCS at 2 weeks follow-up** | | | | |
| --- | --- | --- | --- | --- |
|  | Total  (n=83) | Open repair  (n=40**) | Laparoscopic repair (n=43**) | *p*-value |
| **Mesh sensation (scale 0-40)**  Mean (SD)  Median (range)  Symptomatic patients* (%) | 9.2 (10.7)  7 (0-36)  72% | 11.8 (11.9)  10 (0-36)  76% | 6.9 (9.2)  3 (0-35)  68% | 0.171 |
| **Pain (scale 0-40)**  Mean (SD)  Median (range)  Symptomatic patients (%) | 10.8 (9.6)  9 (0-35)  86% | 12.0 (10.0)  10 (0-33)  93% | 9.7 (9.3)  7 (0-35)  80% | 0.475 |
| **Activity limitation (scale 0-35)**  Mean (SD)  Median (range)  Symptomatic patients (%) | 9.4 (9.8)  6 (0-30)  80% | 11.3 (10.1)  9 (0-30)  88% | 8.0 (9.5)  5 (0-30)  74% | 0.329 |
| **Cumulative CCS score (scale 0-115)**  Mean (SD)  Median (range)  Symptomatic patients (%) | 30.1 (28.5)  24 (0-100)  89% | 35.5 (30.8)  32.5 (0-99)  94% | 25.5 (26.4)  19 (0-100)  85% | 0.307 |

Abbrevations: CCS = Carolina Comfort Scale

* Total scores exceeding 1 were considered symptomatic (ranging from ‘mild but bothersome’ to disabling symptoms)

** 5 patients (4 in the open and 1 in the laparoscopic group) had a non-mesh repair and did not complete the CCS
