# Supplementary material for: The INCH-trial: a multicenter randomized controlled trial comparing short- and long-term outcomes of open and laparoscopic surgery for incisional hernia repair
Source: Surg Endosc. 2023 Oct 9;37(12):9147–58. doi: 10.1007/s00464-023-10446-7 (PMC10709221; doi:10.1007/s00464-023-10446-7)
Supplement: Supplementary file 2 — Supplementary file2 (DOCX 22 KB) [file 464_2023_10446_MOESM2_ESM.docx]

**SUPPLEMENT**

| **Supplement 2. Postoperative quality of life score by CCS at 3 months follow-up** | | | | |
| --- | --- | --- | --- | --- |
|  | Total  (n=18) | Open repair  (n=9) | Laparoscopic repair (n=9) | *p*-value |
| **Mesh sensation (scale 0-40)**  Mean (SD)  Median (range)  Symptomatic patients* (%) | 9.6 (11.5)  3 (0-37)  56% | 7.9 (8.5)  5 (0-22)  67% | 11.2 (14.2)  0 (0-37)  44% | 0.554 |
| **Pain (scale 0-40)**  Mean (SD)  Median (range)  Symptomatic patients (%) | 8.3 (9.5)  3 (0-23)  61% | 9.4 (8.6)  7 (0-22)  89% | 7.11 (10.7)  0 (0-23)  44% | 0.616 |
| **Activity limitation (scale 0-35)**  M Mean (SD)  Median (range)  Symptomatic patients (%) | 6.7 (7.6)  3 (0-22)  72% | 6.1 (7.6)  3 (0-22)  67% | 7.22 (8.0)  3 (0-20)  78% | 0.767 |
| **Cumulative CCS score (scale 0-115)**  Mean (SD)  Median (range)  Symptomatic patients (%) | 24.5 (24.7)  16.5 (0-66)  83% | 23.4 (21.2)  20 (0-66)  89% | 25.6 (29.0)  4 (0-65)  78% | 0.862 |

Abbrevations: CCS = Carolina Comfort Scale

*Total scores exceeding 1 were considered symptomatic (ranging from ‘mild but bothersome’ to disabling symptoms)
