# Supplementary material for: The INCH-trial: a multicenter randomized controlled trial comparing short- and long-term outcomes of open and laparoscopic surgery for incisional hernia repair
Source: Surg Endosc. 2023 Oct 9;37(12):9147–58. doi: 10.1007/s00464-023-10446-7 (PMC10709221; doi:10.1007/s00464-023-10446-7)
Supplement: Supplementary file 3 — Supplementary file3 (DOCX 22 KB) [file 464_2023_10446_MOESM3_ESM.docx]

**SUPPLEMENT 3**

| S3. Postoperative quality of life score by CCS at 5 years follow-up | | | | |
| --- | --- | --- | --- | --- |
|  | Total  (n=50) | Open repair  (n=29) | Laparoscopic repair (n=21) | *p*-value |
| Mesh sensation (scale 0-40)  Mean (SD)  Median (range)  Symptomatic patients* (%) | 3.2 (5.8)  0 (0-23)  19/50 (38%) | 3.8 (6.8)  0 (0-23)  10/29 (35%) | 2.3 (3.9)  0 (0-13)  9/21 (43%) | 0.367 |
| Pain (scale 0-40)  Mean (SD)  Median (range)  Symptomatic patients (%) | 4.9 (8.1)  0 (0-32)  24/50 (48%) | 6.17 (9.5)  1 (0-32)  16/29 (55%) | 3.1 (5.5)  0 (0-21)  8/21 (38%) | 0.182 |
| Activity limitation (scale 0-35)  Mean (SD)  Median (range)  Symptomatic patients (%) | 4.5 (7.2)  0 (0-28)  22/50 (44%) | 5.1 (7.7)  1 (0-28)  15/29 (52%) | 3.8 (6.8)  0 (0-20)  7/21 (33%) | 0.523 |
| Cumulative CCS score (scale 0-115)  Mean (SD)  Median (range)  Symptomatic patients (%) | 12.6 (19.4)  0 (0-83)  29/50 (58%) | 15.1 (22.3)  1 (0-41)  18/29 (62%) | 9.1 (14.4)  1 (0-83)  11/21 (52%) | 0.288 |

Abbrevations: CCS = Carolina Comfort Scale

*Total scores exceeding 1 were considered symptomatic (ranging from ‘mild but bothersome’ to disabling symptoms)
