# Supplementary material for: The INCH-trial: a multicenter randomized controlled trial comparing short- and long-term outcomes of open and laparoscopic surgery for incisional hernia repair
Source: Surg Endosc. 2023 Oct 9;37(12):9147–58. doi: 10.1007/s00464-023-10446-7 (PMC10709221; doi:10.1007/s00464-023-10446-7)
Supplement: Supplementary file 4 — Supplementary file4 (DOCX 21 KB) [file 464_2023_10446_MOESM4_ESM.docx]

**SUPPLEMENT**

| **Supplement 4. Recurrence rates at 5 years follow-up** | | | | |
| --- | --- | --- | --- | --- |
|  | Total  (n=68) | Open repair  (n=37) | Laparoscopic repair (n=31) | *p*-value |
| Recurrence, n (%)  No recurrence, n (%) | 13 (19%)  55 (81%) | 6 (16.2%)  31 (83.8%) | 7 (22.6%)  24 (77.4%) | 0.251 |
